# Food insecurity and the use of coping strategies on multimorbidity, anxiety and depression in South African adults: A nationally representative study

**DOI:** 10.1371/journal.pone.0340695

**Published:** 2026-01-09

**Authors:** Olatundun Gafari, Ashleigh Craig, Khuthala Mabetha, Duncan Hornby, Craig Hutton, Mary Barker, Shane A. Norris

**Affiliations:** 1 School of Primary Care, Population Sciences and Medical Education, University of Southampton, Southampton, United Kingdom; 2 NIHR Southampton Biomedical Research Centre, University Hospital Southampton NHS Foundation Trust and University of Southampton, Southampton, United Kingdom; 3 SAMRC/Wits Developmental Pathways for Health Research Unit, Faculty of Health Sciences, University of the Witwatersrand, Johannesburg, South Africa; 4 DSI-NRF Centre of Excellence in Human Development, University of the Witwatersrand, Johannesburg, South Africa; 5 Sustainability and Resilience Institute, University of Southampton, Southampton, United Kingdom; 6 Geodata Institute, University of Southampton, Southampton, United Kingdom; 7 School of Health Sciences, University of Southampton, Southampton, United Kingdom; 8 School of Human Development and Health, University of Southampton, United Kingdom; 9 Institute for Life Sciences, University of Southampton, Southampton, United Kingdom; Indian Institute of Information Technology, INDIA

## Abstract

**Objective:**

To assess the associations between food insecurity, coping strategies, socio-economic status and anxiety, depression and multimorbidity in South Africa.

**Methods:**

Data from a nationally representative cross-sectional survey conducted in April 2024 (n = 3171; weighted to 20,955,234 adults aged > 18 years) were used. Food insecurity was measured using the Community Childhood Hunger Identification Project (CCHIP) tool, a validated household-level measure commonly used in South Africa. Coping strategy, anxiety and depression were measured using the coping strategies index, Generalised Anxiety Disorder–7 scale and Patient Health Questionnaire–9, respectively. Multimorbidity was self-reported as ≥2 of 14 known chronic conditions. Multivariable logistic regression was used to test associations, and a generalised structural equation model examined the roles of socio-economic status and coping strategies.

**Results:**

Being from a food-insecure household more than doubled the odds of experiencing multimorbidity (OR=2.17, 95% CI 2.17, 2.19), depression (OR=2.96, 95% CI 2.95, 2.97) and anxiety (OR=2.82, 95% CI 2.81, 2.83). Food insecurity accounted for approximately 60% of the total association between socio-economic status and depression, and about 88% of the association between socio-economic status and multimorbidity.

**Conclusions:**

Food insecurity is significantly associated with adverse physical and mental health outcomes. Interventions to improve food security, especially in low socio-economic populations, should be prioritised given their associations with multimorbidity, anxiety and depression. Potential intervention effects will require longitudinal or experimental evaluation.

## Introduction

Food insecurity, a situation when “a person lacks regular access to enough safe and nutritious food to ensure normal growth and development, and an active and healthy life” [1], has negative implications for both physical and mental health. Together with its consequences, food insecurity disproportionately affects low-resource and vulnerable settings, particularly affecting countries in Africa, including South Africa. For example, it was reported that three in every five people were found to be food insecure in Africa in 2022 [1] compared to a global prevalence of three in ten people [1]. These negative impacts of food insecurity, coupled with the recognition of food access as a fundamental human right [2] and how it is linked with all the United Nations Sustainable Development Goals [1,3], makes food insecurity a matter of growing public health significance.

In South Africa, food insecurity continues to remain a significant public health challenge, affecting 20.4% of households in 2021 [4] and 23.7% in 2022 [5]. This problem is further exacerbated by local vulnerabilities, including high levels of inequalities, high unemployment rates, poor access to healthcare services, rising cost of living and slow recovery from the COVID-19 pandemic [6–8].

Previous studies have shown an association between food insecurity and a range of negative health outcomes, including chronic diseases, incidence of infectious diseases and multimorbidity [9], an important measure of physical health [10]. Multimorbidity is defined as the co-existence of two or more long-term conditions in the same individual [11], resulting in reduced quality of life, overburdened health systems and economic losses [12]. In South Africa, a country with the highest global burden of HIV and a growing burden of non-communicable diseases, multimorbidity poses a serious concern [13], resulting in reduced quality of life, increased demand on an already stretched healthcare system and increased mortality [13,14]. Although there is growing evidence suggesting that food insecurity may contribute to the onset of multimorbidity, this relationship remains underexplored in South Africa.

In addition to physical health, food insecurity has also been consistently linked with poor mental health outcomes, including anxiety and depression [4,5]. A previous study using a nationally representative survey found the prevalence of probable anxiety and depression in South Africa to be at about 18% and 26% respectively [15]. Understanding factors that are associated with both multimorbidity, anxiety and depression is therefore important to ensure targeted interventions can be developed to address these problems.

Although some studies highlight the role of food insecurity in the relationship between socio-economic disadvantage and poor mental or physical health [16,17], evidence from low- and middle-income settings remains limited and often does not capture the complex vulnerabilities and coping strategies that shape these pathways [18,19]. Households will often adopt different strategies to either cope with or prevent food insecurity. While these strategies may provide short-term relief, they may also negatively impact on physical and mental health in the long term [4,20].

In South Africa, where both multimorbidity and mental health disorders are on the rise, understanding the complex interactions between food insecurity, coping mechanisms, and health outcomes is vital. We hypothesise that socio-economic and demographic factors, particularly in South Africa where there is a high level of socioeconomic inequalities, will influence the kinds of coping strategies households will adopt and consequently, how those coping strategies will affect the health and wellbeing of the household and its members. Understanding the way that households respond to these fluctuations in food supply and access will enable food system actors and policy makers to develop effective strategies to promote flexibility in their management of future food crises. There is, however, a paucity of nationally representative studies examining these relationships, particularly among adults [5]. Most existing studies are regional, population-specific, or lack consideration of the mediating and moderating roles of socio-economic status and coping strategies.

Utilising a nationally representative survey, this study addressed these gaps and aimed to: 1) determine the prevalence of household food insecurity in 2024 and how it has changed since 2021 as well as, documenting coping strategies currently in use in South Africa; 2) assess the impact of food insecurity and coping strategies on multimorbidity, anxiety and depression; and 3) examine how food insecurity and coping strategies mediate the relationship between socio-economic status and multimorbidity, anxiety, and depression.

## Methods

### Study design and setting

This study is a repeated cross-sectional survey of a nationally representative sample of the South African population conducted in April 2024. It follows two previously conducted nationally representative cross-sectional panels in October 2021 (Panel 1; n = 3402) [15] and June 2022 (Panel 2; n = 3459) [5]. For clarity, this cross-sectional panel from which this study is based is hereafter referred to as Panel 3.

**Fig 1:**
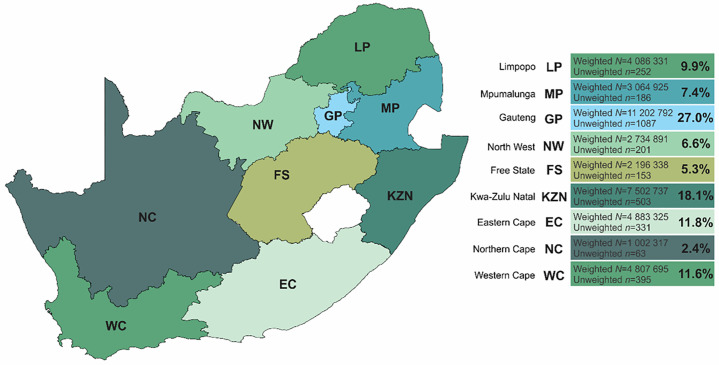
Population demographics and survey distribution of the nine provinces of South Africa [21].

Through a six-stage stratified probability sampling, further described elsewhere [4], 3171 adults aged >18 years were interviewed through an interviewer administered questionnaire in April 2024 across the nine provinces of South Africa (Fig 1). In summary, in the first stage, community sizes including metropolitan areas, cities, towns, large villages, and rural areas, and gender distributions were identified to ensure proportional representation across provinces. The second stage involved the random selection of small geographic areas as primary sampling units, with a minimum of six interviews conducted in each. In the third stage, Geographic Information System (GIS) mapping was used to determine a random starting point within every selected area. The fourth stage entailed household selection, beginning at the identified starting point and proceeding systematically so that every sixth household was approached. The South African definition of a household as individuals living together for at least four nights per week and sharing common resources was adopted. During the fifth stage, interviewers documented the total number of households within each dwelling, after which a built-in randomization program selected specific households to be interviewed. Finally, in the sixth stage, an automated Kish grid was used to randomly select one eligible adult respondent (aged ≥18 years) within the chosen household. Recruitment to the study was conducted from the 9^th^ of March to the 8^th^ of April 2024.

### Data collection

An international research company, IPSOS (www.ipsos.com), carried out data collection activities on behalf of the researchers using the same procedures as in Panels 1 and 2, explained elsewhere [15,22]. In summary, trained and experienced fieldworkers went to households across the nine provinces of the country to administer the survey through face-to-face interviews using a computer-assisted personal interviewing technology. To ensure generalisability, a community-targeted approach was used to facilitate enrolment within each community.

### Survey questionnaire

The survey questionnaire included questions on the respondent’s age, sex, employment status, level of education, marital status, urbanicity (metropolitan, city/town, rural/village), mental health and health conditions. Information was also collected on household assets, food insecurity level and food-related coping strategies.

### Measurement of predictor: food insecurity

Food insecurity was assessed using an adapted version [23] of the validated Community Childhood Hunger Identification Project (CCHIP) tool [24,25]. The CCHIP tool is a measure that has been validated and widely used in South African national surveys to measure household-level food insecurity [26]. It includes three questions related to food insecurity experienced in the past 12 months, namely:

1) ‘Does your household ever run out of money to buy Food?’
2) ‘Do you ever cut the size of meals or skip meals because there is not enough money for food?’
3) ‘Do you or any of your children ever go to bed hungry because there is not enough money to buy food?’

Respondents who answered ‘no’ to all three questions were given a score of 0 and classified as ‘Food Secure’ households. Those who answered ‘Yes’ to only one of the three questions were given a score of 1 and were classified as ‘At risk’, while those who answered ‘Yes’ to two or three questions were given a score of 2 or 3, respectively and were classified as ‘Food Insecure’ [4].

### Measurement of outcomes: anxiety, depression and multimorbidity

Both the GAD-7 and the PHQ-9 have been validated for use in South Africa [27,28]. The Generalised Anxiety Disorder (GAD-7) scale was used to assess anxiety [29]. The scale is made up of seven questions about symptoms of anxiety, with a four-point Likert response scale ranging from 0 (‘Not at all’) to 3 (‘nearly every day’). Level of anxiety was categorised into two groups: a GAD-7 score of 10 or more was defined as probable anxiety and a GAD-7 score less than 10 was defined as no anxiety [30]. The Patient Health Questionnaire (PHQ-9) scale consisting of nine questions assessing different symptoms of depression with a four-point Likert scale ranging from 0 (‘Not at all’) to 3 (‘nearly every day’) was used to assess depression [29,31]. Respondents with a PHQ-9 score of 10 or greater were defined as having probable depression [31].

Respondents were asked health related questions relating to the 14 known chronic conditions in South Africa (heart attack, stroke, high cholesterol, diabetes, overweight/obesity, HIV/AIDS, asthma/chronic obstructive pulmonary disease, sore joints/muscle problems (i.e., arthritis, gout), tuberculosis, cancer, liver disease, mental health (i.e., depression, anxiety, bipolar), chronic kidney disease and hypertension/high blood pressure). A multimorbidity score was calculated by summing the number of chronic conditions. Respondents with two or more chronic conditions were classified as having multimorbidity. Others were classified as not multimorbid.

### Measurement of confounding variables: coping strategies and socio-economic status

Household asset score between one and 21 was used as a measure of socio-economic status (SES) in this study, aligning with the Demographic and Health Survey’s household questionnaire. Households were given a score based on the number of major operational household amenities or assets, e.g., refrigerator, tumble dryer, vacuum cleaner, they had from a list of 21 items [22].

The Coping Strategies Index tool was used to measure strategies for coping with food insecurity [32]. This standard questionnaire, consisting of 11 food-related coping strategies, assesses how households cope during periods of limited food access. Respondents are asked to choose between one of the following frequency options (‘Less than once a week’, ‘1–2 times per week’, ‘3–6 times per week’, ‘Everyday’ and ‘Never’), indicating how often they use each strategy.

### Data analysis

Data analyses were conducted using IBM’s Statistical Package for the Social Sciences (SPSS) version 29 and Stata version 18.0. QGIS was used to scale and plot the geography of South Africa (Fig 1). Using a random iterative method [33], all models and statistics were weighted to represent the most recent census of the South African population (Census 2022; n = 20,955, 234; aged ≥18years old). Weighting factored age, gender, home language, ethnicity and provincial distribution.

Univariate and multivariable binary logistic regression were carried out to determine the association of food insecurity on anxiety, depression and multimorbidity, while adjusting for SES, age, sex, marital status, education, employment and urbanicity as confounders. These confounders were selected apriori based on existing evidence linking them to both exposure and outcomes and contextual knowledge of the South African population. Due to the large sample size, we interpreted findings based on odds ratios and 95% confidence intervals, rather than relying solely on p-values [34,35]. Mental health conditions were excluded from the multimorbidity score in analyses involving anxiety and depression to avoid multicollinearity.

A generalised structural equation model (gSEM) was used to examine the direct and indirect relationships between food insecurity, multimorbidity, anxiety and depression and the role SES and coping strategies played in these relationships. The choice of gSEM was based on the need to assess these complex associations involving multiple outcomes and mediators. A principal component analysis was conducted using the 11 coping strategy variables to generate factor variables for coping strategy. Components with an eigenvalue >1 were retained. Independent interpretable factors were obtained using varimax rotation and factor loading of ≥0.10 was applied to interpret the factor patterns. Double loading was handled by placing the variable in the component with the strongest loading factor. Only factor scores with a cumulative percentage >50 were used in further analyses. Two-factor variables were generated from the principal component analyses (S1 Appendix) and the factor with the higher number of variables was used to indicate coping strategy in the gSEM.

Direct (unmediated), indirect (mediated) and total effects were computed using non-linear combination estimates and the proportion of total effect mediated was calculated by dividing the coefficients of the indirect effects by the total effect. Modifications to pathways were done in an iterative manner. The Akaike and Bayesian Information Criteria (IC) of each model were compared and the final model was selected if it had a low IC and high theoretical relevance. Respondents with missing variables for either food insecurity, SES, coping strategy, anxiety, depression and multimorbidity were excluded and data for 1,745 respondents were used in the analyses. The gSEM was conceptualised before data analyses began.

### Ethics

This study was conducted according to the guidelines laid down in the Declaration of Helsinki and all procedures involving research study participants were approved by the Human Research Ethics Committee (Non-medical) (H21/06/36), University of Witwatersrand, South Africa. Written informed consent was obtained from all study respondents.

### Inclusivity in global research

Additional information regarding the ethical, cultural, and scientific considerations specific to inclusivity in global research is included in the Supporting Information (S2 Appendix).

## Results

### Sample demographics

Characteristics of the study sample are presented in Table 1 by food insecurity groups. There were 3171 respondents in the study sample, just over half of whom were women (50.4%). When the sample was weighted to the South African population, most respondents were within the 25–34-year-old age bracket (27.8%). Only about half of the respondents had completed secondary school education or equivalent (49.7%) and were employed (44.3%). Most respondents were single (57.5%) and lived in metropolitan areas (41.9%) compared to city/town (27.7%) or rural/village (30.5%) areas. Respondent demographics were congruent with the sample from the first two waves. The prevalence of probable anxiety and depression and multimorbidity in the population was 13.7%, 21.6% and 16.2% respectively.

**Table 1:** Characteristics of the study sample by food insecurity groups.

Variables	Unweighted					Weighted				
	n = 3171	n = 1745								
%	Total	Food secure n = 854	At risk n = 301	Food insecure n = 590	P values	Total	Food secure	At risk	Food insecure	P values
**Age category**					**0.003**					**<0.001**
18-24 years	18.6	19.8	20.9	16.8		15.1	15.7	18.9	12.6	
25-34 years	29.6	27.4	28.2	29.8		27.8	27.6	28.5	26.8	
35-44years	26.2	31.0	24.2	23.4		22.7	27.6	18.9	21.5	
45-54 years	14.6	14.2	16.9	17.1		14.6	13.9	17.8	17.4	
55-64 years	6.9	5.0	7.0	7.5		12.0	9.6	11.1	13.2	
>65years	4.1	2.6	2.7	5.4		7.8	5.7	4.9	8.6	
**Sex**					0.940					**<0.001**
Male	49.6	42.6	41.5	42.0		47.7	41.6	39.4	39.1	
Female	50.4	57.4	58.5	58.0		52.3	58.4	60.6	60.9	
**Education**					**<0.001**					**<0.001**
No education/Partial primary	1.9	0.8	1.3	3.7		3.6	1.4	2.7	6.4	
Primary	2.5	2.1	3.0	3.2		3.8	3.8	4.7	4.3	
Partial secondary	26.3	19.7	31.6	38.3		27.8	21.8	32.1	40.4	
NSC/Short course	53.1	56.4	52.2	45.8		49.7	53.8	49.7	41.1	
Tertiary	16.2	21.0	12.0	9.0		15.1	19.3	10.8	7.9	
**Employment status**					**<0.001**					**<0.001**
Unemployed	39.4	31.2	45.9	57.3		39.2	33.5	46.0	56.1	
Employed	47.2	56.6	41.2	30.0		44.3	52.7	37.9	27.9	
Student	7.5	9.1	7.6	4.9		6.1	7.1	7.2	3.4	
Retired	5.9	3.2	5.3	7.8		10.4	6.7	8.8	12.5	
**Marital status**					0.073					**<0.001**
Single	62.9	54.9	57.5	61.0		57.5	49.5	55.4	55.7	
Married/Cohabit	30.6	40.3	36.9	32.9		33.0	43.0	36.2	35.3	
Widowed/Divorced/Separated	6.5	4.8	5.6	6.1		9.5	7.6	8.4	9.0	
**Socio-economic status (household assets)**					**<0.001**					**<0.001**
Lower tertile	33.0	23.3	34.9	45.1		36.2	27.8	37.2	49.0	
Middle tertile	39.6	39.7	41.2	40.2		38.8	38.5	44.7	37.6	
Upper tertile	27.4	37.0	23.9	14.7		25.0	33.7	18.2	13.4	
**Urbanicity**					**0.001**					**<0.001**
Metropolitan	53.3	54.2	50.2	48.3		41.9	42.6	37.2	37.2	
City/Town	21.1	18.7	17.9	26.9		27.7	24.9	25.1	31.3	
Rural/village	25.6	27.1	31.9	24.7		30.5	32.5	37.7	31.5	
**Anxiety categories**					**<0.001**					**<0.001**
No anxiety	85.3	90.3	87.4	77.6		86.3	90.9	89.7	78.0	
Probable anxiety^a^	14.7	9.7	12.6	22.4		13.7	9.1	10.3	22.0	
**Depression categories**					**<0.001**					**<0.001**
No depression	77.5	84.2	76.4	65.4		78.4	85.1	75.9	65.9	
Probable depression^b^	22.5	15.8	23.6	34.6		21.6	14.9	24.1	34.1	
**Multimorbidity**					**<0.001**					**<0.001**
0-1 morbidity	86.7	91.1	83.7	79.0		83.8	88.1	81.5	77.3	
2 + morbidities	13.3	8.9	16.3	21.0		16.2	11.9	18.5	22.7	

^a^Probable anxiety was defined as respondents with a GAD-7 score>10. ^b^Probable depression was defined as respondents with a PHQ-9 score>10. Abbreviations: *n*: number of participants; NSC: national senior certificate signifying completed secondary education.

Having a tertiary education, being employed and being in a higher tertile of SES were statistically significantly more common (p < 0.001) among those in the food secure households compared to those in food insecure households. Compared to the food secure and at-risk households, more people in the food insecure households had probable anxiety, depression and multimorbidity and these differences were statistically significant (p < 0.001).

### Food insecurity prevalence and trend in South Africa

**Fig 2:**
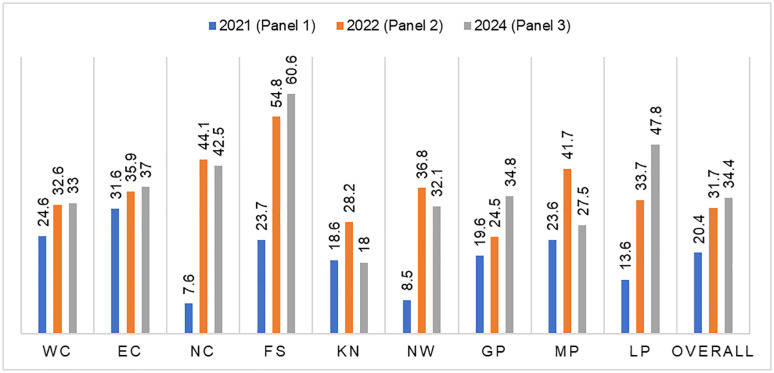
Prevalence and trend of food insecurity across South African provinces. Abbreviations: NC – Northern Cape, WC – Western Cape, NW – Northwest, GP – Gauteng province, LP – Limpopo province, MP – Mpumalanga province, FS – Free State, KN – Kwa-Zulu Natal and EC – Eastern Cape. Numbers presented are percentages of food insecurity.

The prevalence of food insecurity in South Africa, across different provinces at three timepoints (2021, 2022 and 2024), is illustrated in Fig 2. This figure utilises data from the current study (Panel 3) and Panels 1 and 2 (Fig 2). Overall, 34.4% of South African households were classified as being food insecure in 2024, an increase from 20.4% in 2021 (Panel 1). As in Panel 2, Free State was the province with the highest rate of food insecurity in 2024. Across all provinces, except for KwaZulu-Natal, there has been an increase in levels of food insecurity compared with those detected in Panel 1, albeit at different levels. However, when compared with Panel 2, some provinces (i.e., Northern Cape, KwaZulu-Natal, North-West and Mpumalanga provinces) had experienced a reduction in their prevalence of food insecurity.

**Fig 3:**
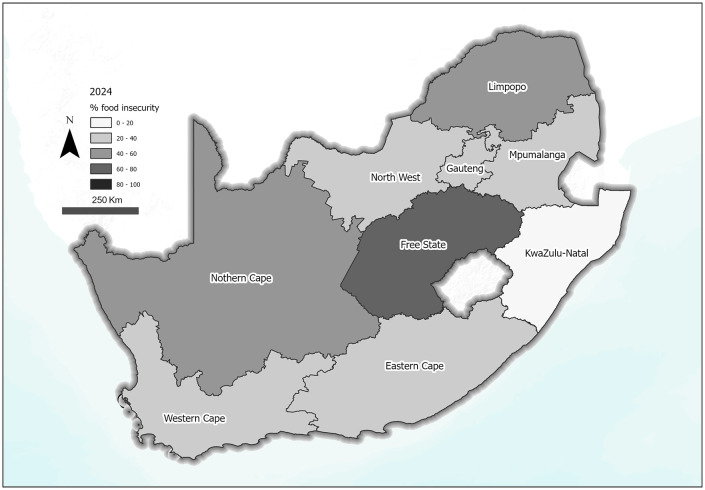
Distribution of food insecurity across South African provinces in 2024. Source: South Africa – Subnational Administrative Boundaries, OCHA, Humanitarian Data Exchange (HDX), licensed under CC BY IGO (https://data.humdata.org/dataset/cod-ab-zaf). Basemap: World Terrain Base, provided by Esri. Data sources include Esri, USGS, NOAA, and other contributors.

**Fig 4:**
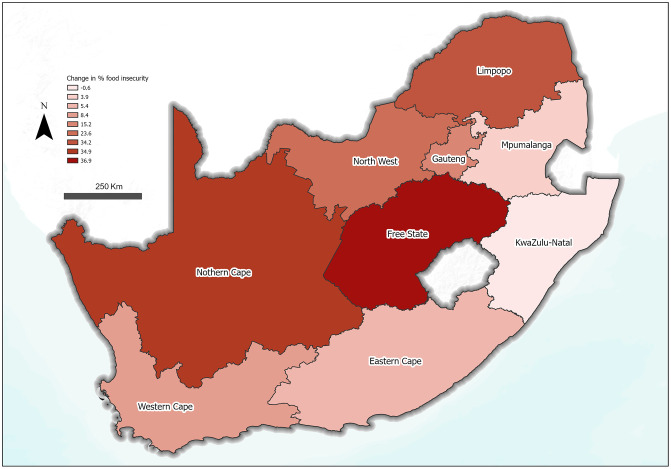
Change in food insecurity prevalence from 2021 to 2024 across the nine South African provinces. Source: South Africa – Subnational Administrative Boundaries, OCHA, Humanitarian Data Exchange (HDX), licensed under CC BY IGO (https://data.humdata.org/dataset/cod-ab-zaf). Basemap: World Terrain Base, provided by Esri. Data sources include Esri, USGS, NOAA, and other contributors.

A graphical representation of the distribution of food insecurity across provinces in South Africa in 2024 is presented in Fig 3. Fig 4 also shows a change map reflecting the differences in food insecurity prevalence between 2021 and 2024 (Fig 4). The figure showed that Free State also had the worst increase (36.9% increase) in food insecurity prevalence from 2021 to 2024 (Fig 4).

### Socio-economic and demographic associations with food insecurity

**Fig 5:**
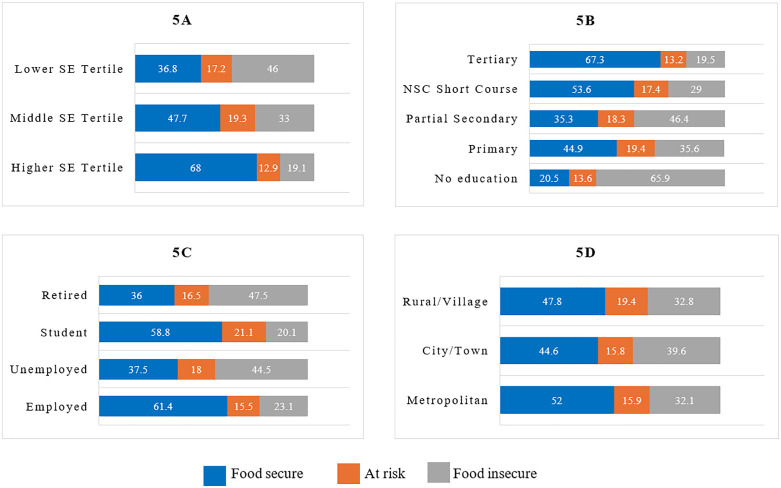
Prevalence of food insecurity by socio-economic and demographic characteristics (a) socio-economic status, (b) education attainment, (c) employment status and (d) urbanicity. Abbreviations: SE: Socio-economic, NSC: National Senior Certificate. Numbers presented are percentages.

The prevalence of food insecurity seen in different socio-economic and demographic groups is presented in Fig 5. The prevalence of food insecurity was higher among retired (47.5%), and unemployed (44.5%) individuals compared with levels amongst those who were employed or students (Fig 5c). Food insecurity prevalence decreased with increasing educational attainment (Fig 5b) and increasing SES (Fig 5a), from 65.9% among those with no education to 19.5% among those with a tertiary education and likewise from 46% among those in a lower SES tertile to 19.1% among those in a higher SES tertile.

### Food-related coping strategies in South Africa

**Fig 6:**
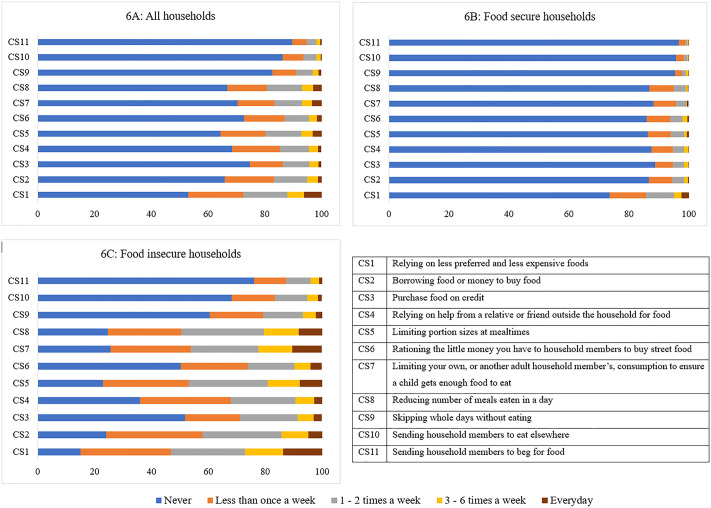
Coping strategies used by South African households.

Fig 6 shows the frequency of use of different coping strategies adopted by South African households to cope with food insecurity. The most used coping strategy was “relying on less preferred and less expensive foods”, with 46.9% of the population using this strategy at least once a week. The least used coping strategy was “sending household members to beg for food” (10.2%). Food-insecure households used all coping strategies more than food-secure households (Fig 6).

### Associations of food insecurity and coping strategies with multimorbidity, anxiety and depression

Table 2 shows the association of food insecurity with multimorbidity, anxiety and depression. The full model statistics are presented in S3 Appendix. Being from a food-insecure household increased the odds (over two times) of experiencing multimorbidity (OR=2.17, 95% CI 2.17, 2.19), probable depression (OR=2.96, 95% CI 2.95, 2.97) and probable anxiety (OR=2.82, 95% CI 2.81, 2.83). These associations were independent of socio-economic status, age, sex, education, employment and urbanicity.

**Table 2:** Associations of food insecurity with multimorbidity, anxiety and depression.

Food insecurity status		Depression			Anxiety			Multimorbidity		
		OR	95% CI	P-value	OR	95% CI	P-value	OR	95% CI	P-value
Model 1	At risk	1.82	1.81, 1.82	<0.001	1.15	1.15, 1.16	<0.001	1.69	1.68, 1.69	<0.01
	Food insecure	2.96	2.95, 2.97	<0.001	2.82	2.81, 2.83	<0.001	2.17	2.17, 2.19	<0.01
Model 2	At risk	1.74	1.733, 1.744	<0.001	1.18	1.175, 1.185	<0.001	1.86	1.857, 1.870	<0.001
	Food insecure	2.56	2.554, 2.566	<0.001	2.62	2.614, 2.629	<0.001	2.18	2.173, 2.185	<0.001

Model 1: Binary logistic regression unadjusted. Model 2: regression adjusted for socio-economic status, age, sex, education, employment and urbanicity. OR: odds ratio.

Table 3 shows the associations of coping strategies with multimorbidity, anxiety and depression. The coping strategy, “sending household members to beg for food” significantly increased the odds of having probable depression (93%) and probable anxiety (89%). Purchasing food on credit was associated with the highest odds of experiencing multimorbidity (OR=1.53, 95% CI 1.52, 1.53).

**Table 3:** Associations of coping strategy with multimorbidity, anxiety and depression.

	Depression			Anxiety			Multimorbidity		
Coping Strategy	OR	95% CI	P-value	OR	95% CI	P-value	OR	95% CI	P-value
1: Relying on less preferred and expensive foods	1.26	1.258, 1.263	<0.001	1.17	1.163, 1.169	<0.001	1.43	1.424, 1.430	<0.01
2: Borrowing food or money to buy food	1.33	1.327, 1.333	<0.001	1.42	1.417, 1.424	<0.001	1.48	1.478, 1.485	<0.01
3: Purchase food on credit	1.34	1.340, 1.345	<0.001	1.10	1.093, 1.098	<0.001	1.53	1.523, 1.529	<0.01
4: Relying on help from relative or friend for food	0.88	0.873, 0.87	<0.001	0.89	0.889, 0.894	<0.001	0.83	0.826, 0.830	<0.01
5: Limiting portion sizes at mealtimes	1.05	1.044, 1.049	<0.001	1.16	1.153, 1.159	<0.001	1.23	1.225, 1.231	<0.01
6: Rationing the money you have for household members to buy street food	1.17	1.169, 1.174	<0.001	0.99	0.987, 0.992	<0.001	1.26	1.252, 1.257	<0.01
7: Limiting your own, or another adult household member’s consumption to ensure a child gets enough food to eat	1.40	1.399, 1.406	<0.001	1.63	1.624, 1.633	<0.001	0.67	0.668, 0.671	<0.01
8: Reducing number of meals eaten in a day	0.98	0.980, 0.985	<0.001	0.87	0.868, 0.873	<0.001	0.95	0.949, 0.955	<0.01
9: Skipping whole days without eating	1.41	1.406, 1.413	<0.001	1.50	1.494, 1.502	<0.001	1.38	1.377, 1.384	<0.01
10: Sending household members to eat elsewhere	0.93	0.928, 0.933	<0.001	0.90	0.893, 0.899	<0.001	1.02	1.013, 1.020	<0.01
11: Sending household members to beg for food	1.93	1.926, 1.937	<0.001	1.89	1.885, 1.898	<0.001	0.82	0.820, 0.826	<0.01

Binary logistic regression unadjusted. Abbreviations: CS – Coping strategy, OR – Odds ratio.

### Mediation effects and interrelationships between food insecurity, coping strategies, socio-economic status, multimorbidity, anxiety and depression

**Fig 7:**
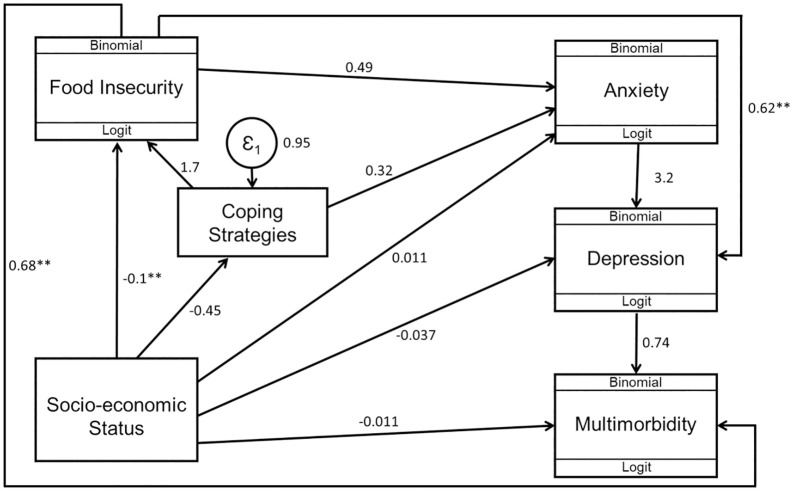
Generalised structural equation model for food insecurity, coping strategies, socio-economic status, multimorbidity, and mental health. Values shown are coefficients of the direct effects. ** p≤0.001.

A gSEM path diagram assessing the relationship between food insecurity, coping strategies, SES, multimorbidity, anxiety and depression was constructed apriori and is presented in Fig 7. The results of the gSEM are presented in Table 4.

**Table 4:** Generalised structural equation model in a sample of respondents for socioeconomic status, food insecurity, coping strategy, anxiety, depression and multimorbidity.

Exposure	Outcome	Total effect		Direct effect		Indirect effect		Proportion of total effect mediated
		Estimate (95% CI)	*p* value	Estimate (95% CI)	*p* value	Estimate (95% CI)	*p* value	
**Effect of SES on depression via Food insecurity**								
SES	PHQ9 < 10	Reference		Reference		Reference		–
	PHQ9 > 10 via food insecurity	−0.10 (−0.148, −0.052)	**<0.001**	–0.037 (−0.076, 0.001)	0.055	−0.06 (−0.099, −0.026)	**0.001**	60%*
**Effect of SES on depression via Anxiety**								
SES	PHQ9 < 10	Reference		Reference		Reference		–
	PHQ9 > 10 (probable)	–0.004 (–0.133, 0.123)	0.957	–0.037 (–0.076, 0.001)	0.055	0.03 (−0.090, 0.157)	0.591	-∞
**Effect of SES on Food Insecurity via Coping strategies**								
SES	Food secure	Reference		Reference		Reference		–
	Food insecure via coping	–0.18 (–0.219, −0.136)	**<0.001**	–0.10 (−0.140, −0.063)	**<0.001**	–0.08 (−0.093, −0.059)	**<0.001**	44.4%^†^
**Effect of SES on multimorbidity via food insecurity**								
SES	0-1 morbidity	Reference		Reference		Reference		–
	2 + morbidities via FI	−0.08 (−0.131, −0.029)	**0.002**	−0.011 (−0.051, 0.029)	0.585	−0.07 (−0.109, −0.029)	**0.001**	87.5%*
**Effect of SES on anxiety via coping strategies**								
SES	GAD7 < 10	Reference		Reference		Reference		–
	GAD7 > 10 via coping	−0.004 (−0.042, 0.035)	0.852	0.011 (–0.028, 0.049)	0.591	–0.014 (–0.021, –0.007)	**<0.001**	-^‡^
**Effect of SES on multimorbidity via depression**								
SES	0-1 morbidity	Reference		Reference		Reference		–
	2 + morbidities via PHQ9 > 10	–0.039 (–0.088, 0.011)	0.126	–0.011 (−0.051, 0.029)	0.585	–0.028 (–0.058; 0.003)	0.074	71.8%∞
**Effect of Food insecurity on multimorbidity via depression**								
FI	0-1 morbidity	Reference		Reference		Reference		–
	2 + morbidities via depression	1.14 (0.754, 1.517)	**<0.001**	0.68 (0.385, 0.976)	**<0.001**	0.45 (0.178, 0.731)	**0.001**	39.5%^†^
**Effect of Food insecurity on depression via anxiety**								
FI	PHQ9 < 10	Reference		Reference		Reference		–
	PHQ9 > 10	2.18 (1.036, 3.318)	**<0.001**	0.62 (0.336, 0.895)	**<0.001**	1.56 (0.455, 2.669)	**0.006**	71.6%^†^

Abbreviations: *n* – number of participants; SES – socioeconomic status; FI – food insecurity; MM – Multimorbidity. *Full mediation, *p* < 0.05; ^†^partial mediation, *p* < 0.05; ^‡^inconsistent mediation, *p* < 0.05; ∞ no mediation.

Food insecurity accounted for approximately 60% of the total association between SES and depression, and about 87.5% of the association between SES and multimorbidity. Furthermore, the analysis showed that about 39.5% of the association between food insecurity and multimorbidity operated indirectly through depression, with the remaining proportion occurring independently. Similarly, the use of coping strategies accounted for approximately 44.4% of the association between SES and food insecurity.

## Discussion

Using a nationally representative panel study of households in South Africa, this study found that the prevalence of food insecurity has increased at a national level, from about 1 in 5 households (20%) in 2021 to almost 2 in 5 (34%) households in 2024. Also, food insecurity is strongly associated with multimorbidity, anxiety and depression, measures of both physical and mental health.

The prevalence of food security reported in the South African National Food and Nutrition Security Survey (NFNSS) conducted in 2023 was higher (63.5%) [36]. A key difference between the surveys that may be responsible for this difference in prevalence is that the two studies used different tools to measure food insecurity. Whereas the CCHIP was used in the study reported in this manuscript, the NFNSS used the Household Food Insecurity Access Scale (HFIAS) [37]. The CCHIP and HFIAS have been validated as good measures of food insecurity in low-income settings [38,39]. While the CCHIP tool categorises households into at-risk or food-insecure groups, the HFIAS tool categorises households into mild, moderate and severe food insecurity groups. This means that some households which would have been classified as having mild or moderate food insecurity using the HFIAS may have only been classified as at risk and not included in the food insecurity prevalence in the current study using the CCHIP tool. A study that compared the use of the CCHIP and HFIAS at overlapping time points found that these tools measured different constructs of food security and so results are not directly comparable [26]. Care, therefore, needs to be taken when comparing findings from the current study with other findings. Ultimately, both studies, regardless of the tools used, showed that food insecurity is significantly higher than they were in 2021.

Reflecting the high levels of inequality in South Africa, the prevalence of food insecurity varied between provinces, with those like Free State presenting with the highest prevalence (60%) and KwaZulu-Natal with the lowest at 18%. Most of the findings by provinces mirror the SES of the different provinces, corroborating the suggestion that SES drives food insecurity. However, the findings for KwaZulu-Natal are unique and contrary to evidence on the drivers of food insecurity. KwaZulu-Natal is known to be one of the poorest provinces in South Africa [40] and in the NFNSS, was among the provinces with the highest prevalence of food insecurity in 2023 [36]. The province also experienced a devastating flood in April 2022, wreaking havoc on lives, economy and livelihoods [41]. However, based on findings from the current study, this province, although it experienced some increase in food insecurity in 2022, had decreased by 2024. KwaZulu-Natal is largely composed of rural land, including areas such as Zululand, where recent government initiatives have encouraged households to grow their own food using available land. As a result, despite persistently high poverty levels, many residents may be less food insecure due to subsistence food production. However, further ground-level research is needed to better understand how communities in KwaZulu-Natal are coping with food insecurity in the context of poverty.

During the time frame covered by the trend analysis in the current study (Fig 2), there were global events such as the COVID-19 pandemic, economic crises and the ongoing Russia-Ukraine war, which resulted in significant impacts on SES and access to food [42,43]. Specific to South Africa were also events such as ‘load shedding’, a euphemism for ‘power cut’, which resulted in electricity shortages and severe flooding affecting parts of the Eastern Cape and KwaZulu-Natal. All these may play a role in the increase in levels of food insecurity in Free State (23.7% to 60.6%), Northern Cape (7.6% to 42.5%) and Limpopo (13.6% to 47.8%) between 2021 and 2024. This highlights previous findings that food systems in many low- and middle-income countries are particularly susceptible to shocks. More ground research is therefore required to understand factors driving this vulnerability, if policies and programmes are going to be successful in reducing the impact of future shocks and crises, including those represented by climate change.

Findings from the current study show food insecurity to be associated with anxiety, depression and multimorbidity independent of SES and other demographic characteristics. In previous studies [44], including a 2019 systematic review of studies from ten countries (low and high-income) [45] and a global analysis of data from 143 countries [46], food insecurity was consistently linked with adverse mental health outcomes including stress, depression, anxiety and psychological distress. Findings also align with other national surveys in South Africa including the National Income Dynamics Study Coronavirus Rapid Mobile Survey (NIDS-CRAM) [8] and Panel 1 of this study published elsewhere [4]. Nationally representative studies assessing the associations between food insecurity and multimorbidity are limited in African countries, though, current findings are similar to those from higher-income nations [9].

There are complex mechanisms that may underlie this association. For instance, as households begin to experience mild food insecurity, coping strategies are initiated, including buying less expensive foods that may be of lower nutritional quality, which was the most used coping strategy by households in the study. This heightens stress and anxiety. The more chronic the implementation of coping strategies and food insecurity becomes, the more anxiety may intensify. A vicious cycle may be established between food insecurity, mental health and multimorbidity since given the potential costs and time, health care access may not be a priority.

Findings from the gSEM also highlight this complex interrelationship between food insecurity, multimorbidity, mental health and SES. The gSEM showed that food insecurity accounted for most (60% and 88% respectively) of the relationship between SES and depression, and SES and multimorbidity. This has significant implications for public health. Prioritising policies and efforts to improve food insecurity especially in low socio-economic populations will be key to reducing the prevalence of multimorbidity, anxiety and depression. Linking nutrition-sensitive programmes such as support for smallholder and subsistence farmers through access to inputs and climate-smart training with mental health and chronic disease services are critical, especially in high-burden provinces like Free State, Eastern Cape, and Limpopo. Targeted efforts toward rural households, informal settlements, and female-headed households could also make national food strategies more equitable and effective [47,48].

## Strengths and limitations

Findings from this study must be interpreted in the context of its strengths and limitations. To the best of the authors’ knowledge, this is the first nationally representative study to assess the prevalence of food insecurity in South Africa in 2024. Trained local field staff led data collection strengthening the quality of the data collected.

A key limitation of the study is the use of self-reported measures of outcome and exposure which may result in recall bias. As this is a cross-sectional study, temporal ordering between exposure and outcome variables cannot be established and reverse or bidirectional relationships may exist. Findings should therefore be interpreted as associations rather than causal effects. Due to the use of the CCHIP tool to measure food insecurity in this study, scope for local and international comparison is limited as the United Nations Food and Agricultural Organisation now recommends the use of the Food Insecurity Experience Scale (FIES). This study, however, provides valuable insights into the state of food insecurity in South Africa in 2024.

## Conclusion

This study reveals that about two in five households in South Africa are food insecure, with the prevalence varying significantly between provinces. The study also highlights the strong associations between food insecurity and physical and mental health. These findings reflect the need for area-based interventions targeted to different communities to improve food insecurity in order to improve both physical and mental health in South Africa.

## Supporting information

S1 Appendix Coping strategy factor scores following principal component analyses. (DOCX)

S2 Appendix Inclusivity in global health research. (DOCX)

S3 Appendix Full regression model statistics. (DOCX)
