# Supplementary material for: Food insecurity and the use of coping strategies on multimorbidity, anxiety and depression in South African adults: A nationally representative study
Source: PLoS One. 2026 Jan 9;21(1):e0340695. doi: 10.1371/journal.pone.0340695 (PMC12788673; doi:10.1371/journal.pone.0340695)
Supplement: S1 Appendix — (DOCX) [file pone.0340695.s001.docx]

**S1 Appendix. Coping strategy factor scores following principal component analyses**

|  | **Factor 1** | **Factor 2** |
| --- | --- | --- |
| Eigenvalue | 4.062 | 2.541 |
| Cumulative % | 58.032 | 63.524 |
| **Coping strategy (CS)** |  | |
| CS 1: Relying on less preferred and expensive foods | 0.447 |  |
| CS2: Borrowing food or money to buy food | 0.583 |  |
| CS3: Purchase food on credit |  | 0.437 |
| CS4: Relying on help from relative or friend for food | 0.528 |  |
| CS5: Limiting portion sizes at mealtimes | 0.692 |  |
| CS6: Rationing the money you have for household members to buy street food | 0.473 |  |
| CS7: Limiting your own, or another adult household member’s consumption to ensure a child gets enough food to eat | 0.640 |  |
| CS8: Reducing number of meals eaten in a day | 0.699 |  |
| CS9: Skipping whole days without eating |  | 0.656 |
| CS10: Sending household members to eat elsewhere |  | 0.737 |
| CS11: Sending household members to beg for food |  | 0.712 |
