# Supplementary material for: Food insecurity and the use of coping strategies on multimorbidity, anxiety and depression in South African adults: A nationally representative study
Source: PLoS One. 2026 Jan 9;21(1):e0340695. doi: 10.1371/journal.pone.0340695 (PMC12788673; doi:10.1371/journal.pone.0340695)
Supplement: S3 Appendix — (DOCX) [file pone.0340695.s003.docx]

**S3 Appendix. Full regression model statistics**

**Table 1. Associations of food insecurity with multimorbidity, anxiety and depression**

|  | **Multimorbidity** | | | **Anxiety** | | | **Depression** | | |
| --- | --- | --- | --- | --- | --- | --- | --- | --- | --- |
|  | **OR** | **95% CI** | **P-value** | **OR** | **95% CI** | **P-value** | **OR** | **95% CI** | **P-value** |
| **Food Insecurity (Ref – food secure)** |  |  |  |  |  |  |  |  |  |
| At risk | 1.863 | 1.857, 1.870 | <0.001 | 1.180 | 1.175–1.185 | <0.001 | 1.739 | 1.733–1.744 | <0.001 |
| Food Insecure | 2.179 | 2.173, 2.185 | <0.001 | 2.622 | 2.614–2.629 | <0.001 | 2.560 | 2.554–2.566 | <0.001 |
| **Gender (Ref Female)** |  |  |  |  |  |  |  |  |  |
| Male | 1.107 | 1.104,1.109 | <0.001 | 1.067 | 1.064–1.070 | <0.001 | 1.028 | 1.026–1.030 | <0.001 |
| **Age (Ref 65+ years)** |  |  |  |  |  |  |  |  |  |
| 18–24 years | 1.319 | 1.312–1.326 | <0.001 | 1.002 | 0.997–1.007 | 0.469 | 1.292 | 1.287–1.297 | <0.001 |
| 25–34 years | 1.957 | 1.947–1.968 | <0.001 | 1.516 | 1.509–1.524 | <0.001 | 1.355 | 1.350–1.361 | <0.001 |
| 35–44 years | 2.538 | 2.524–2.552 | <0.001 | 1.505 | 1.498–1.513 | <0.001 | 1.495 | 1.488–1.501 | <0.001 |
| 45–54 years | 2.948 | 2.931–2.966 | <0.001 | 1.708 | 1.699–1.718 | <0.001 | 1.405 | 1.398–1.411 | <0.001 |
| 55–64 years | 6.871 | 6.822–6.920 | <0.001 | 1.916 | 1.901–1.931 | <0.001 | 1.936 | 1.924–1.949 | <0.001 |
| **Education (Ref Tertiary)** |  |  |  |  |  |  |  |  |  |
| No education/Partial primary | 1.237 | 1.229–1.245 | <0.001 | 1.714 | 1.698–1.730 | <0.001 | 0.955 | 0.948–0.962 | <0.001 |
| Primary | 0.305 | 0.303–0.306 | <0.001 | 1.774 | 1.761–1.787 | <0.001 | 1.518 | 1.510–1.527 | <0.001 |
| Partial secondary | 0.267 | 0.265–0.268 | <0.001 | 1.610 | 1.598–1.623 | <0.001 | 1.057 | 1.051–1.063 | <0.001 |
| NSC/Short course | 0.447 | 0.444–0.450 | <0.001 | 1.900 | 1.884–1.916 | <0.001 | 1.049 | 1.042–1.056 | <0.001 |
| **Employment (Ref Retired)** |  |  |  |  |  |  |  |  |  |
| Employed | 0.893 | 0.890–0.895 | <0.001 | 1.018 | 1.015–1.021 | <0.001 | 1.106 | 1.103–1.109 | <0.001 |
| Unemployed | 1.494 | 1.484–1.504 | <0.001 | 0.825 | 0.819–0.831 | <0.001 | 0.673 | 0.669–0.678 | <0.001 |
| Student | 0.978 | 0.973–0.983 | <0.001 | 0.785 | 0.780–0.790 | <0.001 | 0.987 | 0.981–0.992 | <0.001 |
| **Urbanicity (Ref Metro)** |  |  |  |  |  |  |  |  |  |
| City/Town | 1.274 | 1.270–1.278 | <0.001 | 1.031 | 1.028–1.034 | <0.001 | 1.106 | 1.103–1.109 | <0.001 |
| Rural/Village | 1.056 | 1.053–1.060 | <0.001 | 0.588 | 0.586–0.591 | <0.001 | 0.961 | 0.958–0.964 | <0.001 |
| **Socioeconomic status (Ref High SES Tertile)** |  |  |  |  |  |  |  |  |  |
| Lower SE tertile | 1.332 | 1.328–1.336 | <0.001 | 0.697 | 0.694–0.699 | <0.001 | 0.857 | 0.855–0.860 | <0.001 |
| Middle SE tertile | 1.349 | 1.343–1.354 | <0.001 | 0.685 | 0.682–0.687 | <0.001 | 0.887 | 0.884–0.890 | <0.001 |
